# Racial differences in comorbidity profile among patients with chronic obstructive pulmonary disease

**DOI:** 10.1186/s12916-018-1159-7

**Published:** 2018-10-04

**Authors:** Hyun Lee, Sun Hye Shin, Seonhye Gu, Di Zhao, Danbee Kang, Yeong Rae Joi, Gee Young Suh, Roberto Pastor-Barriuso, Eliseo Guallar, Juhee Cho, Hye Yun Park

**Affiliations:** 1 Division of Pulmonary Medicine and Allergy, Department of Internal Medicine, Hanyang University College of Medicine, Seoul, South Korea; 2 Division of Pulmonary and Critical Care Medicine, Department of Medicine, Samsung Medical Center, Sungkyunkwan University School of Medicine, Seoul, South Korea; 3 Center for Clinical Epidemiology, Samsung Medical Center, Seoul, South Korea; 4 Department of Clinical Research Design and Evaluation, SAIHST, Sungkyunkwan University, Seoul, South Korea; 5 Department of Epidemiology and Welch Center for Prevention, Epidemiology, and Clinical Research, Johns Hopkins University Bloomberg School of Public Health, Baltimore, MD USA; 6 National Center for Epidemiology, Instituto de Salud Carlos III, and Consortium for Biomedical Research in Epidemiology and Public Health (CIBERESP), Madrid, Spain

**Keywords:** COPD, Comorbidity, Race, Ethnicity

## Abstract

**Background:**

Chronic obstructive pulmonary disease (COPD) is often accompanied by multiple comorbidities, which are associated with an increased risk of exacerbation, a poor health-related quality of life, and high mortality. However, differences in comorbidity profile by race and ethnicity in COPD patients have not been fully elucidated.

**Methods:**

Participants aged 40 to 79 years with spirometry-defined COPD from the U.S. National Health and Nutrition Examination Survey (NHANES) (2007–2012) and from the Korea NHANES (2007–2015) were analyzed to compare the prevalence of comorbidities by race and ethnicity group. Comorbidities were defined using questionnaire data, physical exams, and laboratory tests.

**Results:**

Non-Hispanic Whites had the highest prevalence of dyslipidemia (65.5%), myocardial infarction (6.2%), osteoarthritis (40.1%), and osteoporosis (13.6%), while non-Hispanic Blacks had the highest prevalence of asthma (24.0%), hypertension (70.2%), stroke (7.3%), diabetes mellitus (DM) (23.3%), anemia (16.4%), and rheumatoid arthritis (11.9%). Compared to non-Hispanic Whites, non-Hispanic Blacks had a significantly higher prevalence of hypertension, stroke, DM, anemia, and rheumatoid arthritis after adjusting for age, sex, body mass index, and smoking status, while Hispanics had a significantly higher prevalence of DM and anemia, and Koreans had significantly lower prevalences of all comorbidities except stroke, DM, and anemia.

**Conclusions:**

COPD-related comorbidities varied significantly by race and ethnicity, and different strategies may be required for the optimal management of COPD and its comorbidities in different race and ethnicity groups.

## Background

Chronic obstructive pulmonary disease (COPD) is a common respiratory disease characterized by persistent respiratory symptoms and airflow limitations [1]. Due to the ongoing epidemic of smoking and to prolonged life expectancy, COPD is projected to increase in prevalence and to contribute over 4.5 million annual deaths worldwide by 2030 [2, 3].

Since aging and smoking are independent risk factors not only for COPD, but also for multiple chronic diseases, COPD is often accompanied by comorbidities, including cardiovascular disease (CVD), osteoporosis, metabolic syndrome, depression, and several types of cancer [4]. Furthermore, in COPD patients, these comorbidities are associated with an increased risk of exacerbation [5], a poor health-related quality of life [6], increased utilization of health services [7], and high mortality [8]. As a consequence, recent COPD guidelines give special emphasis to the management of comorbid conditions in COPD [1].

COPD guidelines, however, have been developed using data primarily from populations of European descent. COPD and associated comorbidities have multiple genetic, behavioral, environmental, and socioeconomic risk factors, which may vary substantially across racial groups, like other diseases [9–11], but differences in comorbidity profile by race and ethnicity in COPD patients have not been fully elucidated. This study aimed to evaluate differences in comorbidity profile by race and ethnicity in subjects with COPD participating in nationally representative surveys from the United States and South Korea.

## Methods

### Participants

**Fig. 1:**
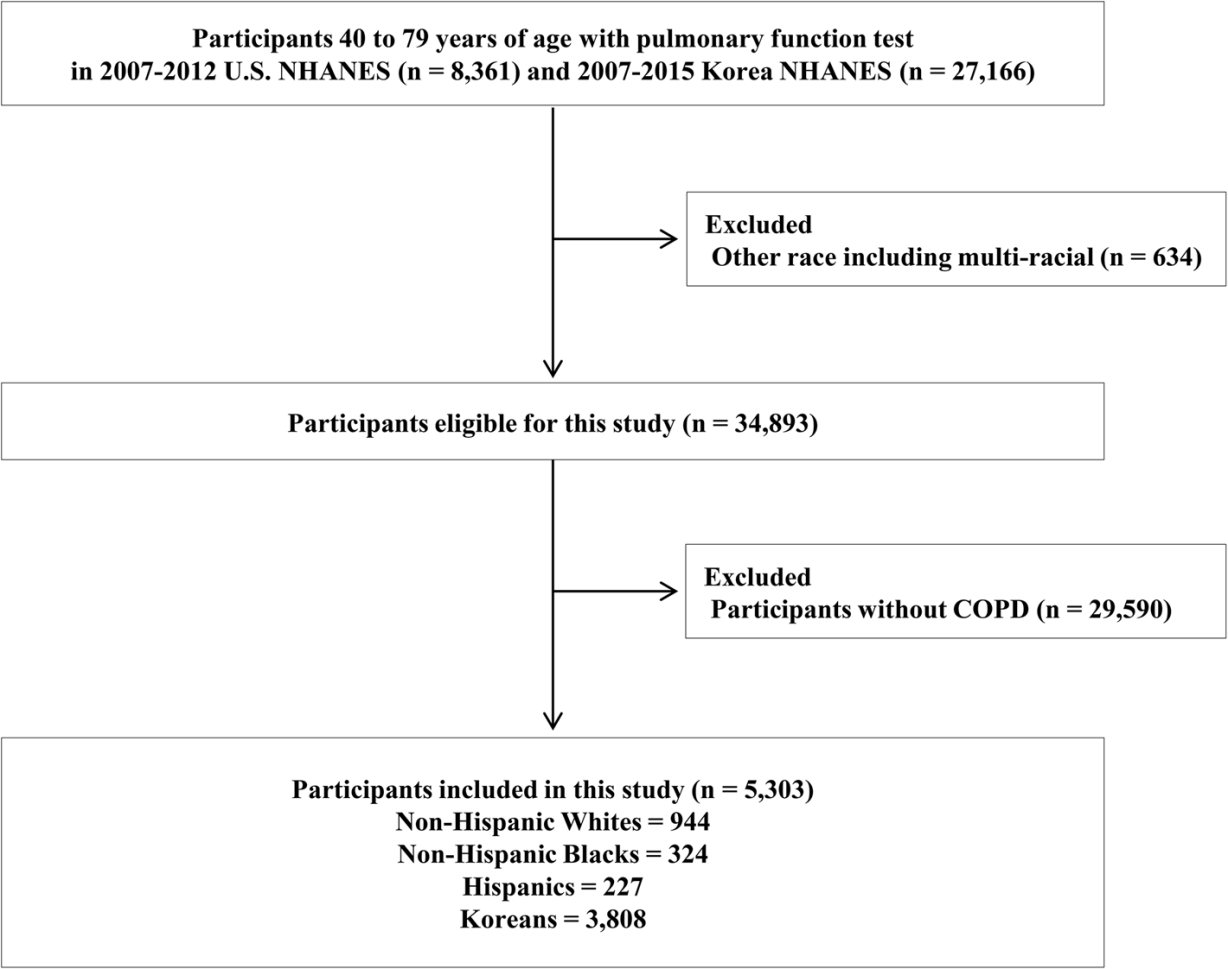
Flow chart of study participants. COPD was defined as pre-bronchodilator forced expiratory volume in 1 s / forced vital capacity < 70%. COPD chronic obstructive pulmonary disease, NHANES National Health and Nutrition Examination Survey

We used data from the 2007–2012 cycles of the U.S. National Health and Nutrition Examination Survey (NHANES) and from the 2007–2015 cycles of the Korea NHANES (KNHANES). Both surveys provide nationally representative data of the non-institutionalized population using a multi-stage cluster sampling design. We restricted our analysis to men and women 40 to 79 years old with spirometry-defined COPD [pre-bronchodilator forced expiratory volume in 1 s (FEV_1_) / forced vital capacity (FVC) < 70%] [12]. Among U.S. NHANES participants, we further restricted the analysis to those who self-identified as Non-Hispanic White (*n* = 944), Non-Hispanic Black (*n* = 324), or Hispanic (*n* = 227). KNHANES provided a representation of Asians (*n* = 3808), as the number of Asian participants with COPD in U.S. NHANES was too small for comparisons (Fig. 1).

### Spirometric measurement

In both surveys, spirometry was performed according to the recommendations of the American Thoracic Society and European Respiratory Society [13]. Absolute values of FEV_1_ and FVC were obtained, and the percentage of predicted values for FEV_1_ and FVC were calculated using the reference equation obtained from an analysis of the general U.S. population for U.S. NHANES [14] and a representative Korean sample for KNHANES [15].

### Definitions

COPD was defined based on pre-bronchodilator FEV_1_ / FVC < 0.7, and COPD severity was classified as mild (FEV_1_ ≥ 80% predicted), moderate (50% ≤ FEV_1_ < 80% predicted), or severe-to-very severe (FEV_1_ < 50% predicted) [1]. Height and weight were measured and body mass index (BMI) was calculated as weight in kilograms divided by height in meters squared. Obesity was defined as BMI ≥ 30.0 kg/m^2^ and overweight was defined as BMI = 25.0–29.9 kg/m^2^ in Non-Hispanic Whites, Non-Hispanic Blacks, and Hispanics, and as BMI ≥ 25 kg/m^2^ for obesity and as BMI = 23.0–24.9 for overweight in Koreans according to World Health Organization guidelines [16].

Comorbidities were defined as a self-reported physician diagnosis. In addition, hypertension was defined as the use of antihypertensive medication, a systolic blood pressure ≥ 140 mmHg, or a diastolic blood pressure ≥ 90 mmHg. Dyslipidemia was defined as the use of lipid-lowering medications or a low-density lipoprotein cholesterol level greater than 130 mg/dL or high-density lipoprotein cholesterol level less than 40 mg/dL [17]. Diabetes mellitus (DM) was defined as the use of glucose-lowering medications or a fasting plasma glucose level ≥ 126 mg/dL. Anemia was defined as hemoglobin level < 13 g/dL in men and < 12 g/dL in women [18].

### Outcomes

We compared the comorbidity profile by race and ethnicity in subjects with COPD participating in U.S. and Korea NHANES. As comorbidities, we included asthma, hypertension, dyslipidemia, stroke, myocardial infarction, DM, and osteoporosis, which were recommended for screening and management in the Global Initiative for COPD guideline [1], plus anemia, osteoarthritis, and rheumatoid arthritis (RA), three common conditions having a substantial impact on the quality of life.

### Statistical analysis

Statistical analysis used the *svy* commands in Stata (release 13.1; StataCorp LP, College Station, TX, USA) to account for survey weights and for the complex sampling design. For each comorbidity, we calculated its prevalence and 95% confidence interval (CI) by race and ethnicity group and used log binomial regression to estimate adjusted prevalence ratios (aPRs) and 95% CIs comparing each race and ethnicity group to Non-Hispanic Whites, adjusting for age, sex, BMI, and smoking status.

## Results

The average age of non-Hispanic Whites, non-Hispanic Blacks, and Hispanics was similar (59.8, 59.6, and 59.6 years, respectively), but Koreans were older on average (63.6 years, *p* < 0.001; Table 1). By sex, the proportions of men among non-Hispanic Whites and non-Hispanic Blacks were similar (59.1 and 60.2%, respectively), and lower than among Hispanic (69.2%) and Korean (73.8%) participants. Hispanic participants were the most likely to be overweight or obese (72.6%), while Non-Hispanic Blacks and Koreans were the most likely to be current smokers (42.4 and 41.6%, respectively). By severity, non-Hispanic Blacks had the highest proportion of severe-to-very-severe COPD (9.2%), while Hispanics had the lowest (3.6%).

**Table 1:** Characteristics of participants with COPD aged 40–79 by race and ethnicity, U.S. NHANES 2007–2012 and Korea NHANES 2007–2015^a^

	U.S. NHANES			Korea NHANES	
	Non-Hispanic White (*n* = 944)	Non-Hispanic Black (*n* = 324)	Hispanic^b^ (*n* = 227)	Korean (*n* = 3808)	*p* value
Age, years	59.8(0.4)	59.6 (0.7)	59.6 (0.8)	63.6 (0.2)	< 0.001
Age group, years					< 0.001
40–49	18.7 (1.7)	20.7 (2.9)	19.7 (3.1)	11.3 (0.7)	
50–59	31.2 (2.2)	30.9 (2.6)	29.9 (3.9)	23.2 (0.9)	
60–69	30.3 (2.4)	24.1 (1.8)	32.0 (3.0)	31.2 (0.9)	
70–79	19.8 (1.3)	24.2 (2.5)	18.3 (2.7)	34.3 (1.0)	
Men, %	59.1 (2.4)	60.2 (2.3)	69.2 (4.2)	73.8 (0.9)	< 0.001
BMI, kg/m^2^	27.7 (0.2)	27.8 (0.4)	28. 6 (0.4)	23.7 (0.1)	< 0.001
BMI group					0.023
Underweight	1.9 (0.5)	3.1 (1.2)	0	2.7 (0.4)	
Normal	32.3 (1.6)	35.1 (2.8)	27.4 (3.4)	38.9 (1.1)	
Overweight	37.8 (1.5)	33.4 (2.3)	41.9 (3.7)	27.6 (0.9)	
Obese	27.9 (1.4)	28.4 (2.6)	30.7 (3.8)	30.7 (1.0)	
Smoking					< 0.001
Current	33.5 (2.4)	42.4 (3.4)	26.4 (2.8)	41.6 (1.0)	
Former	39.9 (2.1)	26.4 (2.2)	35.5 (2.9)	27.9 (0.9)	
Never	26.6 (2.1)	31.3 (2.8)	38.1 (3.3)	30.5 (1.0)	
Spirometry					
FVC, % predicted	96.5 (0.7)	96.7 (1.1)	97.9 (1.4)	90.5 (0.3)	< 0.001
FEV_1_, % predicted	80.3 (0.7)	77.5 (1.1)	82.6 (1.3)	77.8 (0.3)	< 0.001
COPD severity^c^					0.106
Mild	52.3 (2.3)	46.5 (3.1)	60.4 (4.1)	46.4 (1.0)	
Moderate	41.3 (2.2)	44.4 (3.1)	36.0 (4.3)	48.7 (1.1)	
Severe-to-very severe	6.4 (0.8)	9.2 (2.3)	3.6 (1.1)	4.9 (0.4)	

^a^Values are presented as weighted means (standard error of the mean) or weighted percentage (standard error of the percentage)

^b^Hispanic was defined as Mexican American or other Hispanic

^c^Participants were categorized as having mild (FEV_1_ / FVC < 0.7 and FEV_1_ ≥ 80% predicted), moderate (FEV_1_ / FVC < 0.7 and 50% ≤ FEV_1_ < 80% predicted), or severe-to-very severe (FEV_1_ / FVC < 0.7 and FEV_1_ < 50% predicted) disease based on the Global Initiative for Chronic Obstructive Lung Disease guideline

*COPD* chronic obstructive pulmonary disease, *NHANES* National Health and Nutrition Examination Survey (NHANES), *BMI* body mass index, *FEV*_*1*_ forced expiratory volume in 1 s, *FVC* forced expiratory vital capacity

**Fig. 2:**
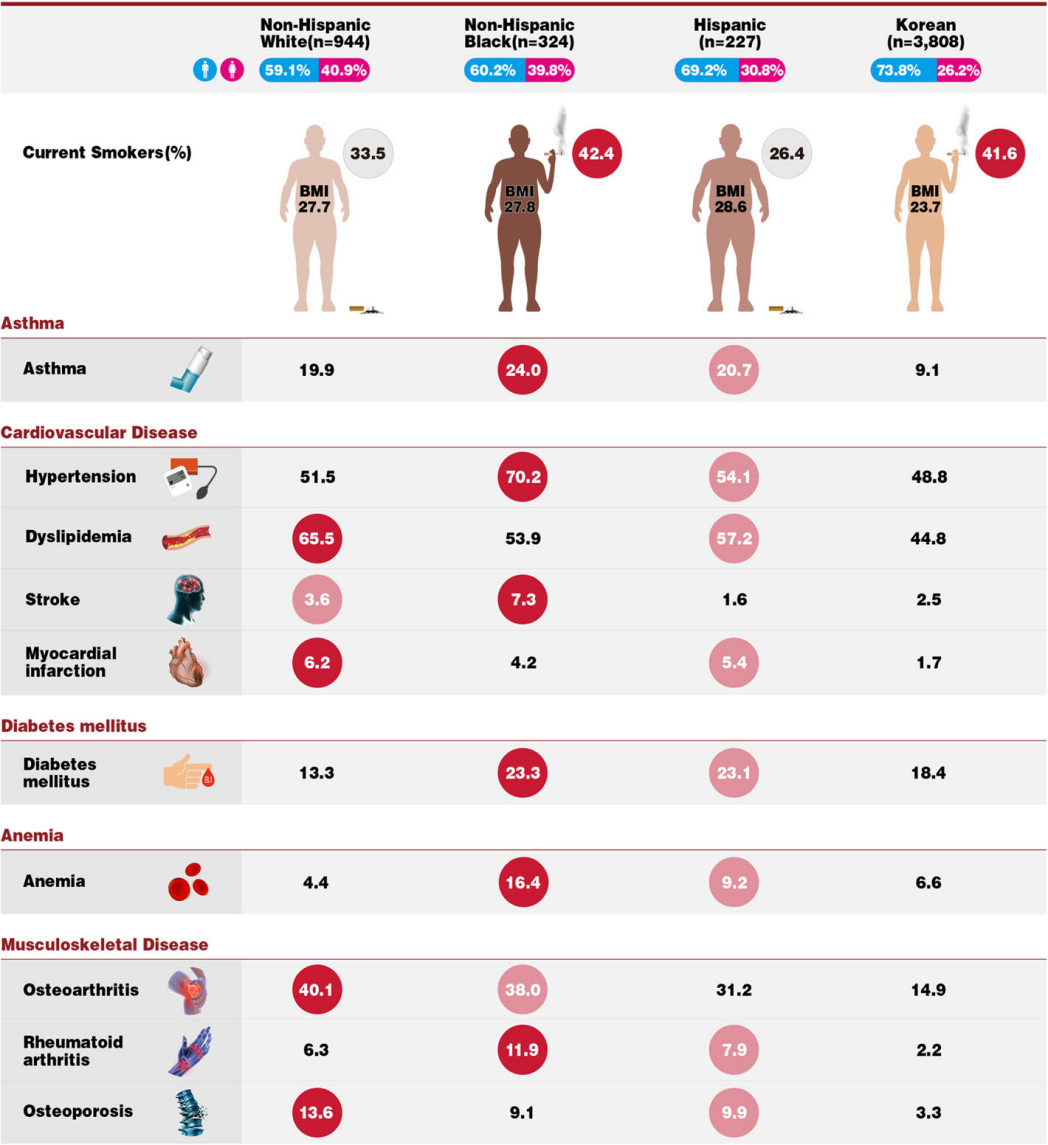
Prevalence of comorbidities among participants with COPD aged 40–79 by race and ethnicity, U.S. NHANES 2007–2012 and Korea NHANES 2007–2015. COPD chronic obstructive pulmonary disease, NHANES National Health and Nutrition Examination Survey

Non-Hispanic White participants had the highest prevalence of dyslipidemia (65.5%), myocardial infarction (6.2%), osteoarthritis (40.1%), and osteoporosis (13.6%), while non-Hispanic Blacks had the highest prevalence of asthma (24.0%), hypertension (70.2%), stroke (7.3%), DM (23.3%), anemia (16.4%), and RA (11.9%) (Table 2 and Fig. 2). Hispanics had very high prevalences of all comorbidities except for stroke, ranking second in the prevalence of asthma, hypertension, myocardial infarction, DM, anemia, RA, and osteoporosis compared to other race and ethnicity groups. Koreans had the lowest prevalence of all comorbidities except for stroke, DM, and anemia.

**Table 2:** Prevalence of comorbidities (95% confidence intervals) among participants with COPD aged 40–79 by race and ethnicity, U.S. NHANES 2007–2012 and Korea NHANES 2007–2015

	U.S. NHANES			Korea NHANES	
	Non-Hispanic White (*n* = 944)	Non-Hispanic Black (*n* = 324)	Hispanic^a^ (*n* = 227)	Korean (*n* = 3808)	*P* value
Asthma	19.9 (16.5 to 23.7)	24.0 (19.4 to 29.3)	20.7 (14.3 to 29.0)	9.1 (8.0 to 10.4)	< 0.001
Cardiovascular disease					
Hypertension	51.5 (47.4 to 55.7)	70.2 (64.0 to 75.6)	54.1 (46.4 to 61.5)	48.8 (46.8 to 50.8)	< 0.001
Dyslipidemia	65.5 (61.2 to 69.7)	53.9 (47.3 to 60.3)	57.2 (48.5 to 65.4)	44.8 (42.7 to 47.0)	< 0.001
Stroke	3.6 (2.6 to 4.8)	7.3 (4.6 to 11.3)	1.6 (0.5 to 5.3)	2.5 (2.0 to 3.1)	0.003
Myocardial infarction	6.2 (4.7 to 8.1)	4.2 (2.6 to 6.6)	5.4 (3.3 to 8.8)	1.7 (1.2 to 2.3)	< 0.001
Diabetes mellitus	13.3 (10.8 to 16.4)	23.3 (18.6 to 28.8)	23.1 (17.5 to 29.7)	18.4 (16.8 to 20.1)	< 0.001
Anemia	4.4 (3.0 to 6.3)	16.4 (12.3 to 21.6)	9.2 (5.8 to 14.3)	6.6 (5.7 to 7.6)	< 0.001
Musculoskeletal disease					
Osteoarthritis	40.1 (36.4 to 44.0)	38.0 (33.0 to 43.2)	31.2 (24.5 to 38.8)	14.9 (13.5 to 16.5)	< 0.001
Rheumatoid arthritis	6.3 (4.3 to 9.1)	11.9 (8.4 to 16.4)	7.9 (5.2 to 12.0)	2.2 (1.6 to 2.8)	< 0.001
Osteoporosis	13.6 (10.9 to 16.9)	9.1 (6.5 to 12.7)	9.9 (6.1 to 15.4)	3.3 (2.7 to 4.1)	< 0.001

^a^Hispanic was defined as Mexican American or other Hispanic

*COPD* chronic obstructive pulmonary disease, *NHANES* National Health and Nutrition Examination Survey

Compared to non-Hispanic Whites, non-Hispanic Black participants had a significantly higher prevalence of hypertension (aPR 1.36, 95% CI 1.23 to 1.51), stroke (aPR 2.01, 95% CI 1.16 to 3.47), DM (aPR 1.76, 95% CI 1.34 to 2.31), anemia (aPR 3.82, 95% CI 2.42 to 6.04), and RA (aPR 1.83, 95% CI, 1.16 to 2.89) after adjusting for age, sex, BMI, and smoking status, while Hispanics had a higher prevalence of DM (aPR 1.64, 95% CI 1.18 to 2.29) and anemia (aPR 2.18, 95% CI 1.23 to 3.86). Koreans had significantly lower prevalences of all comorbidities except stroke, DM, and anemia (Table 3).

**Table 3:** Adjusted prevalence ratios and 95% confidence intervals for comorbidities in participants with COPD aged 40–79, U.S. NHANES 2007–2012 and Korea NHANES 2007–2015^a^

	U.S. NHANES			Korea NHANES
	Non-Hispanic White (*n* = 944)	Non-Hispanic Black (*n* = 324)	Hispanic^b^ (*n* = 227)	Korean (*n* = 3808)
Asthma	Reference	1.23 (0.92 to 1.65)	1.05 (0.72 to 1.53)	0.56 (0.43 to 0.72)
Cardiovascular disease				
Hypertension	Reference	1.36 (1.23 to 1.51)	1.03 (0.89 to 1.20)	0.88 (0.81 to 0.95)
Dyslipidemia	Reference	0.83 (0.72 to 0.96)	0.86 (0.73 to 1.00)	0.68 (0.63 to 0.73)
Stroke	Reference	2.01 (1.16 to 3.47)	0.47 (0.14 to 1.61)	0.69 (0.45 to 1.06)
Myocardial infarction	Reference	0.66 (0.39 to 1.12)	0.86 (0.49 to 1.53)	0.21 (0.14 to 0.33)
Diabetes mellitus	Reference	1.76 (1.34 to 2.31)	1.64 (1.18 to 2.29)	1.11 (0.90 to 1.38)
Anemia	Reference	3.82 (2.42 to 6.04)	2.18 (1.23 to 3.86)	1.31 (0.90 to 1.91)
Musculoskeletal disease				
Osteoarthritis	Reference	0.95 (0.82 to 1.10)	0.81 (0.65 to 1.01)	0.35 (0.30 to 0.40)
Rheumatoid arthritis	Reference	1.83 (1.16 to 2.89)	1.40 (0.85 to 2.31)	0.31 (0.20 to 0.50)
Osteoporosis	Reference	0.68 (0.45 to 1.03)	0.74 (0.46 to 1.20)	0.22 (0.16 to 0.32)

^a^Adjusted for age, sex, BMI group (underweight, normal, overweight, or obese using World Health Organization criteria for the U.S. population and Asian criteria for the Korean population), and smoking status (current, former, or never)

^b^Hispanic was defined as Mexican American or other Hispanic

*COPD* chronic obstructive pulmonary disease, *NHANES* National Health and Nutrition Examination Survey

## Discussion

We found major differences in the comorbidity profile among race and ethnicity groups in subjects with COPD. Non-Hispanic Whites had a comorbidity pattern characterized by dyslipidemia, myocardial infarction, osteoarthritis, and osteoporosis. Non-Hispanic Blacks had a high prevalence of current smokers as well as a high prevalence of multiple comorbidities, especially asthma, hypertension, stroke, DM, anemia, and RA. Hispanics had the highest average BMI levels, as well as high prevalences of asthma and DM. Finally, Koreans had the highest prevalence of current smokers, but lower prevalences of other comorbidities except for stroke, DM, and anemia. Given that coexisting comorbidities have an adverse impact on COPD prognosis, early recognition and management of prevalent disease based on racial differences could reduce the clinical burden of disease in COPD patients.

CVD is a key comorbidity in COPD patients and a major determinant of mortality and functional status [19]. In our study, non-Hispanic Whites had a pattern of CVD comorbidities characterized by a high prevalence of dyslipidemia and coronary disease, while non-Hispanic Blacks had a pattern driven by hypertension, DM, and stroke. These were like the results of a previous study that compared COPD comorbidities between non-Hispanic Whites and non-Hispanic Blacks [20]. In comparison, Hispanics had a pattern driven by obesity and DM. In addition to CVD, asthma is a major comorbidity as well as a risk factor in COPD, complicating treatment and further impairing functional status. Asthma prevalence was particularly high among non-Hispanic Blacks, although non-Hispanics Whites and Hispanics also had a relatively high prevalence. Considering their high prevalence and disease burden, clinicians should consider active screening and management of CVD and asthma in COPD patients.

Anemia has recently been recognized as an independent prognostic predictor of increased hospitalization and mortality in COPD [21–24]. It is also an important comorbidity linked to nutritional deficiency and poor exercise performance among COPD patients [23]. Previous studies have suggested the possibility of improving functional outcomes by correcting anemia [25, 26], and active screening and management of anemia among COPD patients may be beneficial for clinical outcomes, especially among non-Hispanic Blacks and Hispanics.

Non-Hispanic Blacks had very high prevalences of COPD risk factors and comorbidities. Multiple sources of health disparities and inequalities, including socioeconomic factors, environmental hazards, behavioral factors, and access to health care and preventive services, contribute to the excess burden of disease and mortality among non-Hispanic Blacks in the U.S. [27]. The presence of multiple comorbidities may further complicate management and prognosis in these patients [28, 29]. It may be necessary, however, to develop integrated approaches to prevention and management to reduce the burden of chronic disease-related morbidity and mortality, particularly among non-Hispanic Black subjects with COPD.

Hispanic subjects with COPD had a very high burden of overweight and obesity and DM, although the prevalence of myocardial infarction was lower than in non-Hispanic Whites. The lower prevalence of CVD, despite relatively high levels of metabolic risk factors among Hispanics, has been termed the Hispanic paradox [30], but the reasons for this paradox are controversial. Irrespective of the implications of elevated BMI in Hispanic patients, the high prevalence of overweight and obesity in this group should prompt proper management, as morbid obesity is a risk for poor management and functional decline in COPD [31, 32].

Finally, although Korean COPD patients were older, more likely to be smokers, and more likely to be male than other race or ethnicity groups, they had the lowest prevalences of most comorbidities. A similar pattern has been observed in other Asian countries [33]. In particular, the low prevalence of myocardial infarction in Koreans may reflect lower background rates of the disease due to environmental or genetic factors. Since smoking is a predominant risk factor for COPD among Asians and considering the increasing number of Asians in the U.S., more active surveillance regarding COPD and its comorbidities may be warranted.

Several limitations need to be considered when interpreting our findings. First, we used a cross-sectional study and we do not have information on the timing of the development of each comorbidity. We were, thus, unable to establish causal inferences*.* Our objective, however, was to compare the comorbidity patterns across race and ethnicity groups, not to identify causal pathways of comorbidities. The different comorbidity patterns may be due to different prevalences of risk factors such as smoking, obesity, socioeconomic status, or environmental exposures, but race and ethnicity may affect the development of comorbidities. Second, we could not evaluate the prevalence of some important COPD-related comorbidities or conditions, such as obstructive sleep apnea or use of long-term oxygen therapy, due to lack of data in either U.S. NHANES or KNHANES. Third, we did not have data that would allow us to evaluate the outcomes of the different comorbidities. This information is important for understanding the impact of the differences in comorbidity profile by race and ethnicity. Given the racial differences in outcomes such as emergency room visits and duration of hospital stays [7], further studies should evaluate the management and outcomes of COPD, including exacerbations and mortality, by comorbidity profile. Fourth, we used data for Koreans as we did not have data for other Asian groups. However, Korean COPD patients had similar overall characteristics as other Asian COPD patients, including Taiwanese, Japanese, or Chinese patients, although the degree of exposure to biomass fuels differed across Asian groups [33].

## Conclusions

In our study, COPD-related comorbidities occurred disproportionally according to race and ethnicity, and different strategies may be required for the optimal management of COPD and its comorbidities for different race and ethnicity groups. Furthermore, the generation of local maps of COPD-related comorbidities may help in planning different strategies for the diagnosis, treatment, and prevention of COPD and its associated comorbidities.

## Abbreviations

aPR: adjusted prevalence ratio
BMI: body mass index
CI: confidence interval
COPD: chronic obstructive lung disease
CVD: cardiovascular disease
DM: diabetes mellitus
FEV_1_: forced expiratory volume in 1 s
FVC: forced vital capacity
KNHANES: Korea National Health and Nutrition Examination Survey
NHANES: National Health and Nutrition Examination Survey
RA: rheumatoid arthritis
